# Telemedicine in primary care of older adults: a qualitative study

**DOI:** 10.1186/s12875-024-02518-x

**Published:** 2024-07-17

**Authors:** Vladimir Khanassov, Marwa Ilali, Ana Saavedra Ruiz, Laura Rojas-Rozo, Rosa Sourial

**Affiliations:** Department of Family Medicine, McGill University, 5858 Ch. de la Côte des Neiges, Montréal, QC H3S 1Z1 Canada

**Keywords:** Telemedicine, Older adults, Family physicians, Primary care

## Abstract

**Background:**

The COVID-19 pandemic changed the healthcare system, leading to the rapid evolution and implementation of telemedicine (TM). TM has the potential to improve the quality of primary health care and increase accessibility for the population. However, its use may represent challenges for older people, as they may have distinct needs from the general population due to age-related changes in perceptual, motor, and cognitive capacities. We, thus, aimed to identify potential facilitators and barriers to TM use in primary care for older adults and develop recommendations accordingly.

**Methods:**

We conducted a qualitative study to explore the challenges associated with TM use among older adults and healthcare professionals (HCPs) in primary care practice. Interviews were conducted with 29 older adults, and three focus groups involving HCPs from four McGill family medicine sites were organized. Employing a hybrid codebook thematic analysis, guided by the Consolidated Framework for Implementation Research (CFIR), we identified facilitators and barriers affecting the optimal use of TM by older adults and HCPs. We synthesized the results from semi-structured interviews and focus groups. These findings were then presented during a deliberative dialogue with eight participants, including family physicians, nurses, a social worker, and a government-level TM expert, to validate our results. The purpose was to gather feedback, identify and refine actionable recommendations. Subsequently, we utilized a thematic analysis using the same codebook to synthesize findings from the deliberative dialogue.

**Results:**

Participants agreed that TM contributed to maintaining the continuity of care and was particularly convenient when there was an existing or established patient-physician relationship or for addressing minor health issues. TM was found to be beneficial for people with limited mobility, reducing their exposure to potentially high-risk environments. However, participants expressed concerns about the lack of visual contact, causing essential details to be overlooked. Additionally, issues related to miscommunication due to language or hearing barriers were identified. HCPs perceived that most older adults did not consider phone consultations a medical act. Participants were open to a hybrid approach, combining in-person consultations and TM, based on their specific health conditions. Building upon these results, we formulated seven key recommendations.

**Conclusions:**

Both older adults and HCPs consider TM a good alternative for accessing healthcare services. To improve the effective use of TM, it’s crucial to advocate for a hybrid approach that integrates both in-person and virtual methods. This approach should actively encourage and support individuals in becoming familiar with technological tools.

**Supplementary Information:**

The online version contains supplementary material available at 10.1186/s12875-024-02518-x.

## Background

The COVID-19 pandemic and necessity to avoid face-to-face contact propelled patients, particularly older adults and HCPs, to adopt virtual care [1–3]. In this context, telemedicine (TM) became a widely used mode of care delivery, limiting exposure and minimizing the risk of infectious transmission. The World Health Organization defines TM as a delivery system of health care services using different modalities embedded in information and communication technologies [4]. 

The COVID-19 pandemic transformed health care systems, motivating telemedicine’s rapid evolution and implementation. A recent report of the American Medical Association anticipates that “after the COVID-19 pandemic, $250 billion in care could shift to telehealth, boosting a new field of research and infrastructure development.” [3] In Canada, 50% of patient visits were virtual, 62% of family physicians stated that it had improved patient care access, and 39% of the older adults opted for TM to save on caregiving arrangements [5, 6]. This shift in practice to incorporate TM into regular care will continue post-pandemic, as TM has proven to be a highly effective and necessary tool in delivering primary care to older adults [1, 7, 8].

Prior to starting our qualitative study, our team conducted an extensive systematic mixed-methods studies review focusing on telemedicine in the primary care of older adults [9]. After analyzing a substantial pool of 3,328 references, we identified 20 relevant studies. Our primary objective was to explore the effects of TM and understand the determinants influencing its use within primary care for older adults. Our comprehensive review yielded several key insights. Notably, it indicated that the application of TM in primary care for older adults generally led to positive experiences, elicited high satisfaction, and generated an interest in alternative healthcare delivery models. However, it is imperative to acknowledge the limitations of scientific literature, primarily attributed to the scarcity of studies in this domain. Our conclusion emphasized the need for further research to assess the effectiveness of telemedicine on clinical outcomes and healthcare service utilization among older adults, which we are committed to exploring in the present study.

In the reviews conducted by Heinzelmann [10] and De Albornoz [1], TM was associated with improvements in quality of life, functional status, and clinical outcomes of older adults in primary care [1, 10, 11]. Indeed, older adults with chronic conditions, who require frequent follow-ups or who have difficulties commuting because of physical disability, geographical dispersion, or work reasons may benefit the most [1, 10, 11]. Older adults’ satisfaction with TM appears to be high due to convenience, comfort with technology, and relationship with the physician [10].

Conversely, the use of TM may represent a challenge for older adults as they may have different needs due to potential changes in perceptual, motor, and cognitive capacities [12]. Technological challenges may involve Internet connection, handling video or phone devices, and audio or video quality. Moreover, telephone consultations are restricted to verbal communication; the physician cannot observe the patient’s body language, expressions, movements, and environment [1]. Furthermore, it may not be effective for less literate older adults, who cannot express their medical condition well over the phone, or with pre-existing health-related conditions, such as vision and hearing problems or cognitive loss [1]. 

Implementation science methods can be leveraged to reduce the evidence-to-practice gap [13–15]. Most implementation research studies first aim to investigate the barriers and facilitators influencing the implementation of a practice, guideline, or policy to then select the implementation strategies (i.e., strategies to implement TM in primary care). [16] However, the determinants of TM use in the primary care of older adults are limited [1, 10, 11].

We thus aimed to assess challenges, examine potential effects of TM use in primary care of older adults, and develop practice- and evidence-based recommendations. Our research questions were: (1) How do older adults and healthcare professionals (HCPs) perceive their experiences with TM use? (2) What are the facilitators and barriers to TM use in the care of older adults? (3) What recommendations can improve TM use for older adults and HCPs?

## Methods

### Study design

We conducted a qualitative study to examine the challenges associated with TM use among older adults and HCPs in primary care practice. Employing a qualitative descriptive approach, we conducted semi-structured interviews and focus groups to explore the experiences of both older adults and HCPs. We utilized a hybrid codebook thematic analysis to synthesize findings gathered from interviews and focus groups [17]. To ensure the reliability of our results, we subjected them to scrutiny by a panel of healthcare experts using a deliberative dialogue [18]. This panel comprised family physicians, nurses, a social worker, and a government-level TM expert. The deliberative dialogue was then analyzed using the same codebook thematic analysis [17].

### Study setting

We conducted individual semi-structured interviews with older adults and focus groups with the HCPs from four McGill University family medicine sites and the Local Community Services Center (CLSC) – Herzl clinic of the Jewish General Hospital, CLSC-Côte-de-Neige, CLSC-Park Extension, and CLSC-Metro. We selected them since they all provided teleconsultations to older adults, expressed their interest in participating in the study, and provided a letter of support.

### Data collection

To conduct semi-structured interviews, we requested each family medicine practice compile a list of older adults, 65 years old and over, who had used TM at least once via teleconsultation from March 1st, 2020, to March 31st, 2021. Then, the research coordinator of each clinic contacted potential participants to inquire if they wanted to participate in a study on TM. If a participant expressed interest, the coordinator sought their permission to share their name and phone number with our research team for further contact. A list of interested older adults was given to the research team, and research assistants contacted them to provide an overview of the study’s specifics. Those who accepted to participate were called to obtain verbal consent. Two trained research assistants (RS, MI) conducted individual semi-structured interviews with older adults via phone between October 8th and December 6th, 2021.

For focus groups, we recruited HCPs (including family physicians, nurses, social workers, and physiotherapists). The principal investigator (VK), who is a family physician, capitalized on his professional network to identify potential participants. Subsequently, he contacted them via email, outlining the study’s objectives. Those who expressed interest in participating received information and a consent form via email. Written consent was obtained via electronic signature due to precautions implemented during the COVID-19 pandemic. Although VK may have had incidental contact with some participants through his clinical and teaching roles at the family medicine clinics that serve as McGill University teaching sites, he did not discuss any research specifics during these encounters or outside of clinical and research activities. To maintain objectivity and minimize bias, focus groups were co-facilitated and analyzed by two research assistants (AS, MI), who were not previously known to the participants. The study protocol, including the recruitment process and the conduct of focus groups, was reviewed and approved by the appropriate ethics review board, ensuring adherence to ethical standards and guidelines. Focus groups were on February 17th, March 24th, and April 28th, 2022, via Zoom (Zoom Video Communications, Inc, San Jose, CA).

We sampled until data saturation, meaning we collected and analyzed data until no more new findings were discovered and further data were judged unnecessary [19]. All interviews and focus groups were conducted in English and/or French. All interviews and focus groups were audio-recorded, and recordings were transcribed verbatim while simultaneously de-identifying any personal identifiers.

### Data analysis

#### Semi-structured interviews and focus groups

Our analysis methodology involved using NVivo 12 software to separately categorize quotes from semi-structured interviews and focus groups [20]. This method ensured the preservation of each data source’s uniqueness, allowing for a comprehensive examination of their individual characteristics. We chose to utilize the Consolidated Framework for Implementation Research (CFIR), developed by Damschroder et al., as our guiding analytical framework [21]. This choice was informed by the framework’s effectiveness in identifying internal and external factors influencing organizational practices, particularly in implementing innovations such as TM for older adults in primary care. The CFIR comprises 39 constructs organized into five domains: intervention characteristics, outer setting, inner setting, characteristics of individuals, and process (Appendix 1) [22]. 

Following a hybrid thematic approach, we initially employed key concepts from the Consolidated Framework for Implementation Research (CFIR) as primary deductive coding categories, forming the basis of our hybrid codebook [17]. The research team collaboratively established operational definitions for each category through discussions, representing deductive coding [17]. Two researchers independently coded the data and then met to discuss and reconcile any differences, ensuring that the transcripts were double-coded and analyzed collaboratively. Strategies were used to ensure the credibility, fidelity, and confirmability of the study, such as obtaining data saturation, triangulating data sources, and triangulating analyses from multiple researchers [23].

To evaluate the impact of each construct on the utilization of TM, our research team applied ratings based on valence and strength [21, 22]. Valence categorizes constructs as positive (facilitators), negative (barriers), or neutral factors, while strength measures the degree of influence. Constructs unanimously identified as barriers by participants were assigned a negative valence, whereas those universally recognized as facilitators were attributed a positive valence. Constructs that lacked clear characterization or were described as both barriers and facilitators received a neutral valence. Strength ratings were determined by the frequency of mentions by participants, with any discrepancies resolved through team consensus.

### Deliberative dialogue

Recognizing the importance of involving public participation, especially in complex issues, we opted for deliberative dialogue [24]. This group-oriented approach is particularly effective in bringing together diverse stakeholders and facilitating the integration and interpretation of scientific and contextual data [18]. This process aids in the development of evidence-based recommendations. Deliberative dialogue encourages the inclusion of both converging and diverging perspectives. This inclusive approach brings together individuals with varying responsibilities and decision-making authority, aligning with our goal of generating actionable recommendations to enhance dementia care for both older adults and healthcare providers [24].

To identify potential participants, we reached out to individuals who had expressed interest in participating in the deliberative dialogues during initial semi-structured interviews and focus groups. We provided them with an email outlining the study’s objectives, and upon their expressed interest, we shared the relevant information and consent form electronically. Written consent was obtained via electronic signature. We conducted the deliberative dialogue on June 9th, 2022, using Zoom. This dialogue was audio-recorded, transcribed verbatim, and de-nominalized simultaneously.

After synthesizing the results from semi-structured interviews and focus groups, we engaged in a codebook thematic analysis. This consistent utilization of the same codebook ensured continuity and alignment across all phases of data analysis, including the subsequent deliberative dialogue. By involving diverse stakeholders, we aimed to ensure a comprehensive understanding of the subject matter. Participants actively shared their perspectives, validating our initial findings and offering nuanced insights. The inclusion of diverse stakeholders in the deliberative dialogue was crucial, as it facilitated the exploration of various viewpoints and experiences related to the utilization of TM in primary care for older adults.

## Results

### Semi-structured interviews and focus groups

We interviewed 29 older adults aged 65 and older from four McGill University family medicine sites. They had approximately 2 to 3 teleconsultations from March 2020 to March 2021. We conducted 3 focus groups with family physicians, nurses, social workers, and physiotherapists (see Table 1).

**Table 1:** Demographic information of older adults and healthcare professionals

	Interviews (29 participants)	Focus group (15 participants)	Deliberative dialogue (8 participants)
Age*			
*65–70*	15	-	-
*71–75*	4	-	-
*76–80*	5	-	-
*81–85*	2	-	-
*86–90*	3	-	-
Gender			
*Male*	11	4	3
*Female*	18	11	5
Site			
*CDN*	6	2	1
*JGH*	-	-	2
*Herzl*	8	6	2
*Metro*	8	-	1
*PEX*	7	7	1
*St. Mary*	-	-	1
Number of teleconsultations*			
*2–3*	8	-	-
*4–5*	13	-	-
*> 5*	8	-	-
Participated in the study as			
*Patient*	29	-	2
*Family physician*	-	9	3
*Nurse*	-	3	1
*Social worker*	-	2	1
*Kinesiology*	-	1	-
*Telemedicine expert*	-	-	1

*Information only collected for interview participants

CDN: CLSC Côte-des-Neiges; JGH: Jewish General Hospital; PEX: CLSC Parc-Extensio

Of the 39 CFIR constructs assessed, seven were identified in four domains, including intervention characteristics, outer setting, inner setting, and characteristics of individuals (see Table 2). One CFIR construct was identified as a negative factor (barrier), three as positive factors (facilitator), and three as mixed (facilitator and barrier). The following section describes barriers and facilitators identified within each CFIR domain.

**Table 2:** Summary of identified Consolidated Framework for Implementation Research constructs with their respective valence of influence, justifications, and illustrative responses from older adults and healthcare professionals regarding the use of telemedicine in the primary care of older adults

Identified constructs per CFIR domain	Valence and strength of influence^a^	Justification		Illustrative responses
		Older adults	Healthcare Professionals	
**Intervention characteristics**				
A. Complexity	X	• Easy to access and use phone/cellphone • No technical issues (phone/cellphone) • Difficulty using video/computer devices, accessing the portal, uploading documents • Challenges with describing health issues	• Difficulty using video/computer devices, accessing the portal, uploading documents	*Older adults: ‘Since it’s physical*,* that’s why I couldn’t describe it properly on the phone*,* I couldn’t explain it properly on the phone’ [P62]* *HCP: ‘[…] It was more often the technical aspect was more the barrier of like firewalls and why isn’t this turning on and like I could never figure that out.’ [FG1]*
**Outer setting**				
A. Patient Needs and Resources	X	• Convenient when there is a previous/established relationship with the HCP • Suitable for minor issues that do not need visual/physical examination • Older patients preferred phone consultations • Not suitable for an initial consultation, complex medical cases, perceived emergency	• Patients prefer phone consultation • Convenient when there is a previous/established relationship with the patient • Perceived that patients did not consider phone consultation as a medical act • Not convenient for patients with hearing loss, a barrier language, or multiple co-morbidities	*Older adults*: *‘I think these telephone visits*,* these telephone consultations are good when it stayed at a very simple level’ [P44]* *‘Not ideal at all. You have to see the doctor when you have a problem […] And if you can’t*,* they can’t see your tumor on the telephone’[P49]* *HCP*: *‘For sure*,* for someone I already know*,* I would agree to do some consultations via telemedicine’ [FG2]* *‘As soon as there is a language barrier*,* as soon as you know French or English is not their first language*,* you lose everything*,* all the other ways of communicating other than just words. So*,* there it becomes I think much more difficult’[FG2]* *‘[…] they just choose the phone now and it’s actually very rare that I have patients choosing Visio over phone.’ [FG2]* *‘I do find that the phone call*,* it lends itself a little bit to something more informal […] I’ve had some patients where I finished the telephone visit with them*,* and then they’ll call back to the secretary and will be like oh*,* there’s something I forgot to tell Doctor […]*,* can [they] call me back? […] it’s not like I just call you back*,* […] you have to make another appointment if you need’[FG2]*
**Inner setting**				
A. Structural Characteristics	+ 1	The healthcare system reorganized fast its way to assist the patients		*Older adult: ‘I think they did remarkably well […] I mean*,* they had to switch from having in-person people to the phones and that anyway*,* because of COVID and I think they came up with their plan rather quickly and implemented it and it worked well. […]’ [P4]*
B. Implementation climate 1. Compatibility	-2	• Implement a web platform to write medical concerns	• Implementation of a web platform to write medical concerns: • Legal implications • Professional liability issues • Organization/management challenges Lack of resources	*HCP*: *‘[…] there’s lots of medical legal implications to something like that*,* going into an online platform’ [FG1]* *‘Why should it be something written or informal*,* […] it makes me feel like I can’t do my job properly because I just have like this*,* like one- or two-line sentence from an email*,* and then I’m supposed to […] do my whole medical thinking process […] based on this minimal information*,* like no*,* it needs to be in [an] appointment […]’ [FG2]*
C. Readiness for Implementation 1. Available Resources	+ 1	• Patients have the technology equipment (phone, cellphone, computer, or tablet) • Presence of a portal – website for patients (appointment updates, documents, information)	• Lack of resources to implement a web platform	*Older adult: ‘I use my computer*,* but it can be done with an iPad or iPhone*,* we all have these devices’ [P26]* *HCP: ‘Unfortunately*,* the resources are not large enough to provide that’[FG1]*
**Characteristics of Individuals**				
A. Knowledge and Beliefs about the Intervention	X	• Knowledge (when to use TM): • Regular follow-up • Triage/preliminary consultation • Advantages: • Contribution to maintaining the continuity of care • No need to commute; save time • Flexible with time and setting • Helpful for persons with limited mobility • More frequent follow-up • Reduce the exposure of older adults to potential high-risk environments • Easier access to the physician • Faster resolution of medical concerns / medical attention • Avoid walk-in clinic for minor issues • Easier process for scheduling an appointment • Disadvantages: • No visual contact (phone consultation): - Lack of non-verbal communication - No physical examination ◊ important details could be missed • Perception that it’s more difficult to make a diagnosis for a physician • Perception that consultation time is shorter	• Knowledge (when to use TM): • Triage/preliminary consultation • Follow-up/check-in • Advantages: • Contribution to maintaining the continuity of care • No need to commute • Flexible with time and setting • Helpful for persons with limited mobility • Reduce the exposure of older adults to potential high-risk environments • Contribute to a more efficient medical practice: - Pre-triage - Results ready for the appointment - Reliable and direct information from the patient - Save time • Maintain and improve physician-patient relationship: - Sense of presence - More communication • Disadvantages: • No visual contact (phone consultation): - Lack of non-verbal communication - No physical examination ◊ important details could be missed • Miscommunication: misheard, language barrier • Patients may not consider phone consultation a medical act • Limitation to do and offered a proper diagnostic and treatment • The consultation time may be longer	*Older adults*: *‘The phone is handy if there are few questions or adjustments with medications […] or follow-up with your tests*,* it’s fine’ [P10]* *‘As I said*,* it saves time*,* it saves money*,* and it’s much more efficient.’ [P50]* *‘The drawback on that is that if you’re missing some of the data that had you been in person*,* the doctor might’ve seen that you didn’t think to mention*,* that’s sort of a drawback on that.’ [P4]* *HCP*: *‘I found was that there’s sort of allowed a triage and allowed all preliminary tests to be done prior to getting him out’ [FG1]* *‘For mobility limitation by doing things for them was great for like from my perspective and their perspective.’[FG1]* *‘I think I ended up seeing some of my older patients a bit more frequently than I would if they were only coming into the clinic’[FG1]* *‘I don’t feel like it in any way damages the doctor patient relationship*,* I feel like it maybe even strengthens it because during this pandemic*,* when people were cut off from so many services*,* they felt at least like their family doctor was there on the phone.’ [FG2]* *‘[…] it was very difficult to understand what was going on with them*,* without anything visual either kind of visually observing the patient or observing their environment or getting some collateral information*,* so there’s probably it was more*,* I think for those isolated individuals it was particularly challenging’ [FG1]*
B. Individual stage of change	+ 1	• We need to get used to phone/video consultations • I will continue using video/phone consultations after the pandemic, if there is a combination of in-person and TM, depending on the health condition • If I do not have a choice, I will use phone/video consultations	• I will continue using video/phone consultations after the pandemic	*Older adult: ‘[…] a combination of phone*,* video and in person*,* I think is like the best solution. Maybe you can start with the phone and if that’s not good enough*,* like switch over to video that so that they could see*,* and if that’s not good enough*,* like the doctor will advise you on whether or not to come in.’ [P4]* *HCP: ‘I will happily continue to do it’[FG1]*

a. The valence and strength of influence of each factor can be interpreted in the following ways: **+ 2** = the construct is **definitely a positive influence** for the use of telemedicine in the primary care of older adults, a facilitating influence in work processes, and/or a facilitating influence in implementation efforts. Interviewees describe explicit examples of how key or all aspects of the construct manifests itself in a positive way; **+ 1** = the construct is a **probably a positive influence** for the use of telemedicine in the primary care of older adults, a facilitating influence in work processes, and/or a facilitating influence in implementation efforts. Interviewees make general statements about the construct manifesting itself in a positive way or there is a mixed effect that is overall positive; **0** = the construct has a **neutral influence** for the use of telemedicine in the primary care of older adults; **X** = the construct has a **mixed influence** for the use of telemedicine in the primary care of older adults, the comments are equally positive and negative; – **1** = the construct is a **probably a negative influence** for the use of telemedicine in the primary care of older adults, an impeding influence in work processes, and/or an impeding influence in implementation efforts. Interviewees make general statements about the construct manifesting itself in a negative way or there is a mixed effect that is overall negative; – **2** = the construct is **definitely a negative influence** for the use of telemedicine in the primary care of older adults, an impeding influence in work processes, and/or an impeding influence in implementation efforts. Interviewees describe explicit examples of how key or all aspects of the construct manifest themselves negatively

### Intervention characteristics

*Complexity* represented a mixed influence on TM use among older adults. Even though older adults and HCPs mentioned that it was easy to access and use their telephone or cell phone for teleconsultations, it was difficult when they had to use video conferencing platforms or computer devices or upload documents. Likewise, some older adults stated that describing and expressing their medical conditions was challenging.

*‘Since it is physical*,* that is why I could not describe it properly on the phone; I could not explain it properly on the phone’ [P62]*.

### Outer setting

*Patient needs and resources* were an important factor and represented a mixed influence on TM use among older adults. TM was considered convenient when there was a previous or established patient-physician relationship and to resolve minor issues that did not implicate a visual or physical examination.

*‘I think these telephone visits*,* these telephone consultations are good when it stayed at a very simple level’ [P44]*.

*‘For sure*,* for someone I already know*,* I would agree to do some consultations via telemedicine’ [FG2]*.

Conversely, it was not convenient for an initial consultation, complex medical cases, or perceived emergencies. Likewise, participants mentioned that it was unsuitable for older adults with hearing loss or a language barrier.

*‘Not ideal at all. You have to see the doctor when you have a problem […] And if you cannot*,* they can’t see your tumor on the telephone’[P49]*.

*‘As soon as there is a language barrier*,* as soon as you know French or English is not their first language*,* you lose everything*,* all the other ways of communicating other than just words. So*,* there it becomes*,* I think*,* much more difficult‘[FG2]*.

Most of the older adults preferred phone calls over video consultations.

*‘[…] they just choose the phone now and it’s actually very rare that I have older adults choosing Video over phone.’ [FG2]*.

Nevertheless, HCP perceived that most of the time, older adults did not consider phone consultations to be a medical act.

*‘I do find that the phone call*,* it lends itself a little bit to something more informal […] I’ve had some older adults where I finished the telephone visit with them*,* and then they will call back to the secretary and will be like oh*,* there is something I forgot to tell Doctor […]*,* can [she] call me back? […] it is not like I just call you back*,* […] you have to make another appointment if you need‘[FG2]*.

### Inner setting

*Structural characteristics* were identified as a facilitator of TM use by older adults. Older adults mentioned that the health care system reorganized quickly to assist older adults through TM.

*‘I think they did remarkably well […] I mean*,* they had to switch from having in-person people to the phones and that anyway*,* because of the COVID-19 pandemic*,* and I think they came up with their plan rather quickly and implemented it*,* and it worked well. […]’ [P4]*.

*Compatibility* was considered a barrier to TM use. Some older adults suggested implementing a web platform to write about their medical concerns; however, the HCP brought into focus impeding factors such as legal implications, professional liability issues, and organizational and management challenges.

*‘[…] there are lots of medical*,* legal implications to something like that*,* going into an online platform’ [FG1]*.

*‘Why should it be something written or informal*,* […] it makes me feel like I cannot do my job properly because I just have like this*,* like one- or two-line sentence from an email*,* and then I am supposed to […] do my whole medical thinking process […] based on this minimal information*,* like no*,* it needs to be in [an] appointment […]’ [FG2]*.

*Available resources* were an important facilitator of TM use; older adults and HCPs were equipped with the necessary technology devices (phone, cellphone, computer, or tablet).

*‘I use my computer*,* but it can be done with an iPad or iPhone; we all have these devices’ [P26]*.

### Characteristics of individuals

*Knowledge and beliefs about the intervention* were distinguished constructs and identified as having a mixed influence on TM use among older adults. Participants stated they would use TM for regular follow-ups, triage, or preliminary consultation.

*‘The phone is handy if there are few questions or adjustments with medications […] or follow-up with your tests*,* it is fine’ [P10]*.

*‘I found was that there is sort of allowed a triage and allowed all preliminary tests to be done prior to getting him out’ [FG1]*.

Both older adults and HCP agreed that the main advantages of using TM were that it contributed to maintaining continuity of care, there was no need to commute, it was flexible with time and setting, and it helped to save time. Furthermore, it was helpful for older adults with limited mobility and reduced their exposure to potential high-risk environments.

*‘As I said*,* it saves time*,* it saves money*,* and it’s much more efficient.’ [P50]*

*‘For mobility limitation by doing things for them was great for like from my perspective and their perspective.’[FG1]*.

They also perceived that the frequency of follow-ups increased.

*‘I think I ended up seeing some of my older adults a bit more frequently than I would if they were only coming into the clinic’[FG1]*.

In the case of the HCP, they mentioned that TM contributed to improving the efficiency of their medical practice by determining if an in-person or telephone consultation was needed. TM was also beneficial to obtaining reliable and direct information from older adults that could not go in person, such as older adults at home care facilities. Likewise, TM contributed to maintaining and improving the physician-patient relationship.

*‘I do not feel like it in any way damages the doctor-patient relationship*,* I feel like it maybe even strengthens it because during this pandemic*,* when people were cut off from so many services*,* they felt at least like their family doctor was there on the phone.’ [FG2]*.

Among the main disadvantages, participants noted the lack of visual contact during phone consultations, impeding non-verbal communication, and possibly missing essential details. Moreover, older adults underscored the importance of active listening during phone consultations. Indeed, older adults and HCPs perceived that diagnosing and offering proper treatment would be more challenging.

*‘The drawback on that is that if you are missing some of the data that had you been in person*,* the doctor might have seen that you did not think to mention*,* that is sort of a drawback on that.’ [P4]*.

*‘[…] it was very difficult to understand what was going on with them*,* without anything visual either kind of visually observing the patient or observing their environment or getting some collateral information*,* so there is probably it was more*,* I think for those isolated individuals it was particularly challenging’ [FG1]*.

*Individual stages of change* were facilitators of TM use. Older adults and HCP agreed that they would continue using TM if it was combined with in-person consultations, depending on the medical condition and issue.

*‘[…] a combination of phone*,* video and in person*,* I think is like the best solution. Maybe you can start with the phone and if that is not good enough*,* like switch over to video that so that they could see*,* and if that is not good enough*,* like the doctor will advise you on whether or not to come in.’ [P4]*.

### Deliberative dialogue

The panel consisted of eight participants, comprising older adults, family physicians, nurses, a social worker, and a government-level TM expert (see Table 1). Following our deliberative dialogue, we identified seven recommendations (see Table 3):

**Table 3:** Summary of recommendations regarding the use of telemedicine in the primary care of older adults based on the results of the deliberative dialogue

Recommendation	Findings
1. Promoting patients’ preferences and discuss disadvantages and advantages.	• Preference of phone consultations: easier to access and use • Faster resolution of medical concerns / medical attention • Avoid walk-in clinic for minor issues • Easier process to schedule appointment • Contribute to a more efficient medical practice • Maintain and improve physician- patient relationship • Different perception regarding the duration of the consultation (patient vs. HCP) • Limitation to do and offered a proper diagnostic and treatment
2. Supporting patients in navigating TM platform as needed and providing assistance to foster patients’ self-efficacy and self-management.	• Difficulty using video/computer devices • User Interface: difficulty accessing the web, tools and documents • Add a chat during video consultation
3. Combining TM and in-person consultation.	*Convenient*: • Previous/stablish patient-physician relationship • Regular follows-up, triage/preliminary consultation, minor issues *Not convenient*: • Initial consultation, complex medical cases or perceived emergency • Patients with hearing loss, barrier language, or multiple co-morbidities
4. Maintaining a clear communication with patients.	• Active listening during the teleconsultation
5. Encouraging leadership-driven TM initiatives.	• Lack of patients’ awareness: perception that phone consultation is not consider a medical act
6. Supporting familiarization with available communication technologies.	• Lack of telemedicine/technology education and training • Lack of literature on: • Technology infrastructure • Medical liability • Practice certification and license • Interface standards • Patients’ resources • Telemedicine system
7. Advocating for further accessibility of technological tools to improve patients’ health	• Having access to a GMF’s website to write medical concerns, and receive adequate advice from the family medicine practice: • Legal implications • Professional liability issues • Organization/management challenges • Lack of resources • Develop a GMF website with medical information for the patients • Have a telephone number to access faster/directly an HCP

The study outlined key recommendations for TM implementation in healthcare. Older adults and HCPs favored phone consultations for their accessibility and ease of use, citing benefits like faster medical attention, scheduling convenience, and improved doctor-patient relationships. However, challenges such as difficulties in using video/computer devices and accessing web tools were noted. TM was found suitable for established patient-physician relationships and regular follow-ups, but less so for complex cases or emergencies. Clear communication, particularly active listening, was emphasized during teleconsultations to compensate for the lack of visual cues. Initiatives to promote TM faced challenges, with older adults sometimes not recognizing phone consultations as formal medical acts. Additionally, there was a need for education and training in telemedicine technologies, along with efforts to enhance accessibility for older adults, albeit with potential legal and organizational challenges.

## Discussion

This qualitative study explored barriers and facilitators to using TM in primary care of older adults and proposed recommendations to primary HCPs. We included older adults and HCP to obtain a range of different perspectives. Older adults and HCPs agreed that the preferred mode of TM was phone-based, as it was easier to access and use. According to the Virtual Care Force Task’s report, most virtual care happens over the telephone [2, 25]. Phone consultations are considered a standard and reliable technology in primary care [1].

Conversely, for older adults, there are additional challenges to implementing TM, such as sensory limitations (e.g., visual and hearing impairments), cognitive (e.g., memory), and functional (e.g., mobility, dexterity) decline. In addition, the lack of technological savviness and limited access to technological equipment were commonly reported as key barriers to TM use [12, 26, 27]. According to Luxton et al. [26], half of the individuals in the video conferencing group reported connectivity issues, and 35.7% of the treatment sessions required a phone call to resolve a technical problem. Indeed, it was found that video conferencing consultation was more likely to be used by younger older adults and physicians with technological knowledge [28]. Nevertheless, these issues may not prevent an adequate clinical assessment and can be as effective as in-person consultation when delivered to suitable older adults [1].

As reported in our findings, TM is more convenient when there is a previous or established patient-physician relationship, for regular follow-ups, triage/preliminary consultations, or to resolve minor urgent care issues. Participants agreed that follow-up visits do not require a comprehensive assessment as an initial consultation, complex medical cases, or perceived emergencies that would likely need an in-person evaluation. These align with findings by Watt et al. [29] and Aliberti et al. [12] that evaluated physicians’ telehealth experiences with older adults.

Regarding the advantages and disadvantages of using TM for the care of older adults, the responses of this study’s participants echoed concerns about a lack of visual contact during phone consultations, which is a significant barrier to using TM. Participants underscored that phone consultation is restricted to verbal communication. The physician cannot observe the non-verbal cues and environment of the older adults, and it is not possible to perform a physical exam. Therefore, essential details could be missed and ultimately impact establishing reliable diagnoses. These are supported by recent studies in which primary care found many barriers to implementing TM for geriatric and chronic disease management [12, 30, 31]. Besides, participants voiced their concerns about language and literacy levels as barriers to using TM. Telephone consultations may be more effective for older adults whose native language is English or French and who have higher literacy and can articulate and express their medical condition comfortably over the phone [12, 27, 31].

Despite these disadvantages, the participants expressed many advantages with TM, including maintaining the continuity of care, saving time, and improving the patient-physician relationship. During the COVID-19 pandemic, where older adults were required to socially distance themselves and be confined at home, TM became a way to socialize and access healthcare. Older adults felt cared for, and HCPs benefited from reaching their older adults [31].

Furthermore, our participants mentioned that TM was helpful for people with limited mobility, being flexible with time and setting, reducing the exposure of older adults to potential high-risk environments, and increasing the number of follow-up visits. Studies on the experience of geriatric care professionals in TM indicated that people with chronic conditions requiring repeated visits to their physicians and older adults with difficulties traveling to their health center would benefit the most from teleconsultations [1, 12, 32]. Indeed, TM allowed older adults to access health care and counseling without the need to leave their homes, reducing the risk of high-risk elderly falls, particularly during winter [12, 33]. On the other hand, HCPs emphasized that TM contributed to a more efficient medical practice. It worked as a pre-triage to decide if an in-person visit or teleconsultation was needed, allowed to request the laboratory or imagery tests and have results ready for the appointment, and helped save time from administrative and accommodation processes.

Another critical point raised in our study was the lack of literature, education, and training on TM and technology for HCPs. Findings from previous studies reflected the remaining gaps in the current literature on the appropriate use of TM to meet older adults and HCPs’ needs adequately [2, 25, 27, 31]. The Virtual Care Task Force’s report re-emphasized the need to “establish a framework for pan-Canadian quality-based virtual care governance” and “ensure that standards set by medical regulators support the provision of competent and safe virtual care [25] Likewise, Ware et al. [34] mentioned that to ensure implementation success, developing clear guidelines for documentation, identifying potential older adults, and setting parameter thresholds are necessary. Indeed, stakeholders were encouraged to address challenges and promote evidence-based use of TM [2, 25, 35]. Recent articles have highlighted the need to create new approaches, such as a ‘good website manner’ to provide virtual care effectively [36]. ‘Website manner’ is defined as the clinician’s ability to transfer relational skills, such as offering comfort, listening attentively, tenderly respecting, and providing an empathic response to older adults via technology [36–38]. Besides that, a 2012 survey of e-health in the undergraduate curricula across Canada’s medical schools identified a lack of a common language for e-health across the faculties. While half of faculties indicated using EMRs and EHRs in teaching, there were no consistent approaches, and faculty resources to support e-health were not developed [39]. Indeed, the Virtual Care Task Force’s report found that the literature and research regarding assessing learners in virtual care settings are very minimal [25].

Thus, our study provides key facilitators and barriers to using TM for the primary care of older adults. Based on these, we propose seven recommendations, taking diverse aspects into account, including the patient’s preference, how to support them in the use of TM, flexible approaches, communication strategies, and advocating for further technology accessibility.

Our study has limitations; we obtained older adults’ and HCPs’ perspectives at one point, and perceptions of telemedicine may change progressively. However, we decided to perform interviews 18 months after the pandemic started, allowing us to recruit participants with more extended TM experience.

Furthermore, due to the COVID-19 pandemic public health measures, we did the interviews remotely by phone or video; this could have limited the participation of those unable to use telemedicine. Nevertheless, having heard participants’ positive and negative experiences and views of HCPs helped us to have a general idea of TM use in the practice.

One strength is how the qualitative findings confirm the results of our systematic review, previously conducted by our research team, from the point of view of different stakeholders [9]. Future studies should continue to improve virtual care, exploring the compensation model, which refers to how healthcare providers are reimbursed for telemedicine services, as a key factor in enhancing accessibility and sustainability [40]. 

## Conclusion

This study served to identify facilitators and barriers to using telemedicine in the primary care of older adults and propose recommendations to HCPs to improve the service. Older adults consider telemedicine a good alternative for accessing healthcare services when provided in a hybrid approach combined with in-person consultations. It would be essential to discuss their preferences, disadvantages, and advantages of using telemedicine to resolve their medical issues. Our results also emphasize the importance of promoting adaptability and supporting older adults in navigating TM platforms, maintaining clear communication, encouraging leadership-driven TM initiatives, supporting familiarization with available communication technologies, and advocating for further accessibility of technological tools.

### Electronic supplementary material

Below is the link to the electronic supplementary material.

Supplementary Material 1

## Data Availability

The datasets analysed during the current study are available from the corresponding author on reasonable request.

## References

[1] Carrillo de Albornoz S, Sia K-L, Harris A (2022). The effectiveness of teleconsultations in primary care: systematic review. Fam Pract.

[2] Force TVCT. Virtual Care in Canada: Progress and Potential. 2022.

[3] Henry TA, After. COVID-19, $250 billion in care could shift to telehealth. AMA Digit. 2020.

[4] Ryu S (2012). Telemedicine: opportunities and developments in member states: report on the second global survey on eHealth 2009 (global observatory for eHealth series. Healthc Inf Res.

[5] Canada CoFPo. Virtual care in the patient’s Medical Home. Mississauga, ON.; 2021.

[6] Lemire F, Slade S (2021). Family physicians and the COVID-19 third wave. Can Fam Physician.

[7] Falk W. The state of virtual care in Canada as of wave three of the COVID-19 pandemic: an early diagnostique and policy recommendations. FPT Virtual Care and Digital Health Table https://www.canada.ca/content/dam/hc-sc/documents/corporate/transparency_229055456/health-agreements/bilateral-agreement-pancanadian-virtual-care-priorities-covid-19/template-wf-report-eng.pdf. 2021.

[8] Merrell RC (2015). Geriatric telemedicine: background and evidence for telemedicine as a way to address the challenges of geriatrics. Healthc Inf Res.

[9] Ilali M, Le Berre M, Vedel I, Khanassov V (2023). Telemedicine in the primary care of older adults: a systematic mixed studies review. BMC Prim Care.

[10] Heinzelmann PJ, Williams CM, Lugn NE, Kvedar JC (2005). Clinical outcomes associated with telemedicine/telehealth. Telemedicine J e-Health.

[11] Ryan P, Kobb R, Hilsen P (2003). Making the right connection: matching patients to technology. Telemedicine J e-health.

[12] Aliberti GM, Bhatia R, Desrochers LB, Gilliam EA, Schonberg MA (2022). Perspectives of primary care clinicians in Massachusetts on use of telemedicine with adults aged 65 and older during the COVID-19 pandemic. Prev Med Rep.

[13] Grimshaw JM, Eccles MP, Steen N, Johnston M, Pitts NB, Glidewell L (2011). Applying psychological theories to evidence-based clinical practice: identifying factors predictive of lumbar spine x-ray for low back pain in UK primary care practice. Implement Sci.

[14] Grimshaw JM, Eccles MP, Lavis JN, Hill SJ, Squires JE (2012). Knowledge translation of research findings. Implement Sci.

[15] Presseau J, Mackintosh J, Hawthorne G, Francis JJ, Johnston M, Grimshaw JM (2018). Cluster randomised controlled trial of a theory-based multiple behaviour change intervention aimed at healthcare professionals to improve their management of type 2 diabetes in primary care. Implement Sci.

[16] French SD, Green SE, O’Connor DA, McKenzie JE, Francis JJ, Michie S (2012). Developing theory-informed behaviour change interventions to implement evidence into practice: a systematic approach using the theoretical domains Framework. Implement Sci.

[17] Xu W, Zammit K (2020). Applying thematic analysis to education: a hybrid approach to interpreting data in practitioner research. Int J Qualitative Methods.

[18] Van de Kerkhof M (2006). Making a difference: on the constraints of consensus building and the relevance of deliberation in stakeholder dialogues. Policy Sci.

[19] Fusch Ph DPI, Ness LR. Are we there yet? Data saturation in qualitative research. 2015.

[20] Zamawe FC (2015). The implication of using NVivo software in qualitative data analysis: evidence-based reflections. Malawi Med J.

[21] Damschroder LJ, Aron DC, Keith RE, Kirsh SR, Alexander JA, Lowery JC (2009). Fostering implementation of health services research findings into practice: a consolidated framework for advancing implementation science. Implement Sci.

[22] Cane J, O’Connor D, Michie S (2012). Validation of the theoretical domains framework for use in behaviour change and implementation research. Implement Sci.

[23] Green J, Thorogood N. Qualitative methods for health research. 2018.

[24] Boyko JA, Lavis JN, Abelson J, Dobbins M, Carter N (2012). Deliberative dialogues as a mechanism for knowledge translation and exchange in health systems decision-making. Soc Sci Med.

[25] Force TVCT. Virtual Care: Recommendations for Scaling Up Virtual Medical Services. 2020.

[26] Luxton DD, Pruitt LD, Wagner A, Smolenski DJ, Jenkins-Guarnieri MA, Gahm G (2016). Home-based telebehavioral health for US military personnel and veterans with depression: a randomized controlled trial. J Consult Clin Psychol.

[27] Hunting G, Shahid N, Sahakyan Y, Fan I, Moneypenny CR, Stanimirovic A (2015). A multi-level qualitative analysis of Telehomecare in Ontario: challenges and opportunities. BMC Health Serv Res.

[28] Hammersley V, Donaghy E, Parker R, McNeilly H, Atherton H, Bikker A (2019). Comparing the content and quality of video, telephone, and face-to-face consultations: a non-randomised, quasi-experimental, exploratory study in UK primary care. Br J Gen Pract.

[29] Watt JA, Fahim C, Straus SE, Goodarzi Z (2022). Barriers and facilitators to virtual care in a geriatric medicine clinic: a semi-structured interview study of patient, caregiver and healthcare provider perspectives. Age Ageing.

[30] Kendzerska T, Zhu DT, Gershon AS, Edwards JD, Peixoto C, Robillard R (2021). The effects of the health system response to the COVID-19 pandemic on chronic disease management: a narrative review. Risk Manage Healthc Policy.

[31] Chen W, Flanagan A, Nippak PM, Nicin M, Sinha SK (2022). Understanding the experience of Geriatric Care professionals in using telemedicine to care for older patients in response to the COVID-19 pandemic: mixed methods study. JMIR Aging.

[32] Smith AC, Gray LC (2009). Telemedicine across the ages. Med J Aust.

[33] Batsis JA, Pletcher SN, Stahl JE (2017). Telemedicine and primary care obesity management in rural areas–innovative approach for older adults?. BMC Geriatr.

[34] Ware P, Ross HJ, Cafazzo JA, Laporte A, Gordon K, Seto E (2018). Evaluating the implementation of a mobile phone–based telemonitoring program: longitudinal study guided by the consolidated framework for implementation research. JMIR mHealth uHealth.

[35] Ohannessian R, Duong TA, Odone A (2020). Global telemedicine implementation and integration within health systems to fight the COVID-19 pandemic: a call to action. JMIR Public Health Surveillance.

[36] Teichert E. Training docs on ‘webside manner’ for virtual visits. Mod Healthc. 2016.

[37] Gonzalez R. Telemedicine is forcing doctors to learn webside manner. WIRED. 2017.

[38] McConnochie KM (2019). Webside manner: a key to high-quality primary care telemedicine for all. Telemedicine e-Health.

[39] Association of Faculties of Medicine of Canada CHI. Environmental Scan of e-health in Canadian Undergraduate Medical Curriculum. 2012.

[40] Raes S, Trybou J, Annemans L (2022). How to pay for telemedicine: a comparison of ten health systems. Health Syst Reform.

